# Preprosthetic vestibuloplasty performed by three different types of lasers versus conventional scalpel

**DOI:** 10.3389/froh.2026.1936637

**Published:** 2026-09-04

**Authors:** Diana Florina Nica, Ciprian Ioan Roi, Mircea Rivis, Virgil-Florin Duma, Cosmin Sinescu, Meda Lavinia Negrutiu, Tudor-Alexandru Popoiu, Maria Cristina Langa, Cristian Zaharia

**Affiliations:** 1 Research Center of Dento-Alveolar Surgery, Anesthesia and Sedation in Dental Medicine, Department I of Anaesthesiology and Oral Surgery, Faculty of Dental Medicine, “Victor Babes” University of Medicine and Pharmacy of Timisoara, Timisoara, Romania; 2 3OM Optomechatronics Group, Department of Measurements and Electro-Optics, Faculty of Electronics, Telecommunications, and Information Technology, Polytechnic University of Timisoara, Timisoara, Romania; 3 Research Center in Dental Medicine Using Conventional and Alternative Technologies, Faculty of Dental Medicine, “Victor Babes” University of Medicine and Pharmacy of Timisoara, Timisoara, Romania; 4 Discipline of Medical Informatics and Biostatistics, Department III of Functional Sciences, Faculty of Dental Medicine, “Victor Babeş” University of Medicine and Pharmacy of Timisoara, Timisoara, Romania; 5 Faculty of Dental Medicine, “Victor Babes” University of Medicine and Pharmacy of Timisoara, Timisoara, Romania

**Keywords:** healing process, laser surgery, postoperative complications, secondary epithelization, total prosthesis, vestibular depth, vestibuloplasty

## Abstract

**Background and objectives:**

A stable maxillary total prosthesis in an edentulous jaw requires a definite amount of ridge height. Deficient vestibular depth in an atrophic maxilla can cause severe functional impairment for patients with total prosthesis. This affects their quality of life, with impact on their general health. Preprosthetic vestibule remodeling could be carried out using conventional surgery or lasers to obtain an appropriate depth. The aim of our study was to compare the clinical results obtained through the conventional approach versus the modern laser method, recording postoperative complications.

**Materials and methods:**

Thirty-six patients were selected for this clinical study, were divided in 3 equal groups, and the vestibuloplasty was performed by conventional surgery on the right side and by laser for the left side of the maxilla. The former served as a Control group. For the latter we utilized 3 types of lasers: for Group 1, the vestibuloplasty was performed with a neodymium:yttrium-aluminum-garnet (Nd:YAG) laser, for Group 2 we utilized a diode laser, and for Group 3 the patients were treated using an erbium-doped yttrium aluminum garnet (Er:YAG) laser. Postoperative complications such as edema, pain, and hemorrhage were recorded during the early healing time.

**Results:**

Six weeks after the surgery the vestibule lengthening was measured for each patient. A higher vestibular deepening was obtained for the conventional surgery compared to that of laser-treated sites immediately after the procedure. At the end of the healing process, wound shrinkage was significantly higher in conventional scalpel procedure compared to the laser groups. There were no significant differences in the healing process related to the type of laser. On the other hand, complications after the surgery were higher in the scalpel group compared to all the laser groups.

**Conclusion:**

These results suggest that laser-based treatments are superior compared to conventional ones for preprosthetic vestibuloplasty. Registry: ClinicalTrials.gov, TRN: NCT07462117, Registration date: 30 April 2018.

## 1 Introduction

Vestibuloplasty is an essential procedure that is performed in cases of severe alveolar atrophy or after large augmentation procedures. It is motivated by the lack of keratinized gingiva and by a shallow vestibule (1). Thus, in the cases of a reduced vestibule, a crestal repositioning of muscles takes place. This is followed by aesthetic issues, a thin mucosal tissue, and a decreased lip mobility. Also, the limited vestibule compromises the optimal retention and the stability of the prosthesis.

As a preprosthetic surgery, vestibuloplasty is routinely performed by scalpel, using conventional techniques. However, correlated with today's growing interest in minimal invasive surgery, lasers have become appropriate tools to perform customary surgical approaches (2). This is due to issues of conventional techniques, as pointed out in the following. Therefore, the scope of the present research is to approach a subject related to such laser procedures, which are of high interest today because of their advantages, as detailed in the following.

There are two procedure directions within the conventional treatment: the first option is vestibuloplasty through a secondary epithelization and the second one is gingival augmentation with grafts (3). The most utilized surgical methods to increase the vestibule height by changing the muscle attachment are the Kazanjian, Clark and Edlan-Mejchar methods, modified later on by Godwin, Trauner, Obwegeser, Steinhauser and Tortorelli (4). The oldest techniques of vestibuloplasty were based on applying epidermal graft or skin graft, with the scope to increase the denture bearing surface area and to stabilize the new vestibule depth (5). Other materials utilized over time for obtaining a stable keratinized tissue have included: meshed skin, collagen matrix, mucosal, dermal and reversed dermal grafts, as well as platelets rich fibrin (PFRF) membranes (6). Most of these techniques require a secondary surgical donor field, which entails great morbidity, increases patient discomfort, and extends the surgical time and the risk of postoperative complications (7).

The new technology that has enabled lasers to perform vestibuloplasty can be performed using various type of devices. The most utilized are Nd:YAG, diode, and Er:YAG lasers (8). All of them offer significant advantages, from which the most important in vestibuloplasty are: the possibility to simplify the surgical steps, an excellent hemostatic effect with a better field visibility, reduced anesthesia requirements, as well as a diminished postoperative sensitivity (9–11). Furthermore, the laser treatment allows for creating a supraperiosteal fenestration on the vestibular aspect of the alveolar ridge. Most important, it is able to achieve a stable result without scar formation (12).

The aim of the present clinical study was to evaluate the advantages of three types of lasers (with different wavelengths) for vestibuloplasty in comparison to conventional surgery. Maxillary edentulous patients with a shallow vestibule, who were complaining of prosthesis instability and low retention were included in the study. The gain in vestibule depth that can be obtained through laser procedures in comparison to conventional surgery must be assessed. Also, pain, discomfort, postoperative bleeding, and scar survey are aspects that must be evaluated. Our working hypothesis was that the laser treatment can impact positively the clinical outcome compared to conventional treatments.

## 2 Materials and methods

### 2.1 Study design

A prospective monocentric observational study was carried out from April 2018 to December 2024 at the Department of Oral and Maxillofacial Surgery, Faculty of Dental Medicine of the “Victor Babes” University of Medicine and Pharmacy of Timisoara, Romania. This study was approved by the Ethics Commission for Scientific Research of the University, following the Ethical protocol of the University, with the CECS Approval nr. 09 (approved date: 2 March 2018), and it was carried out according to the Declaration of Helsinki. The study was registered at ClinicalTrials.gov, with the following identification number: NCT07462117.

### 2.2 Dimension of sample and inclusion criteria

Systemically healthy 36 edentulous patients (12 males and 24 females), aged between 62 and 79 years old were included in this prospective, randomized, controlled, comparative clinical study with a partial split-mouth design, as each patient received a laser intervention on one side and a scalpel on the other, but different patients were treated with a different type of laser. We selected the patients to be candidates for total prosthesis and complete edentulous at the level of the maxilla. Gender and age are not important for this type of study, only the comorbidities which were excluded at the time of patient^'^s selection. The randomization into groups was performed manually. The cases that were considered suitable for surgery were subjects with inappropriate vestibular depth where an instability of the prosthesis was anticipated or patients who addressed to the clinic because of a compromised prosthetic support.

Concerning the allocation concealment, we tried, during the study, to equalize the study groups and to move forward with roughly the same number of patients for each of the three considered laser treatments.

Regarding the dimension of the considered sample, a number of more than 10 patients for each group, undergoing the same intervention, can yield relevant results regarding to the wound healing process, from the surgeons' point of view. On the other hand, throughout the study period, the aim was to recruit as many subjects as possible to undergo this type of intervention, in order to obtain reliable and convincing results. To the best of our knowledge, after reviewing the literature, our study includes the largest number of patients who have received the same type of intervention for the same type of edentulism. We must point out that we conducted a *post-hoc* power analysis using an *F*-test for one-way ANOVA, considering the four independent groups (i.e., conventional, diode, Er:YAG, and Nd:YAG). Assuming a medium effect size (f = 0.25), an alpha level of 0.05, and a total sample size of 48 participants (≈12 per group), the achieved statistical power was estimated at approximately 25.4%. This indicates a limited probability of detecting true group differences at this effect size, suggesting that the study may be underpowered. Future studies with larger samples are warranted to increase statistical sensitivity, particularly for detecting subtle differences between surgical methods.

All patients displayed an inadequate vestibular depth and insufficient attached gingiva in the antero-lateral maxilla. They all underwent clinical and radiological examination before surgery. The inclusion criteria were: generally healthy patients, no preventive anticoagulants or antiplatelet therapy, maxillary edentulous, no previous vestibuloplasty, and signed consent provided by every subject.

**Figure 1:**
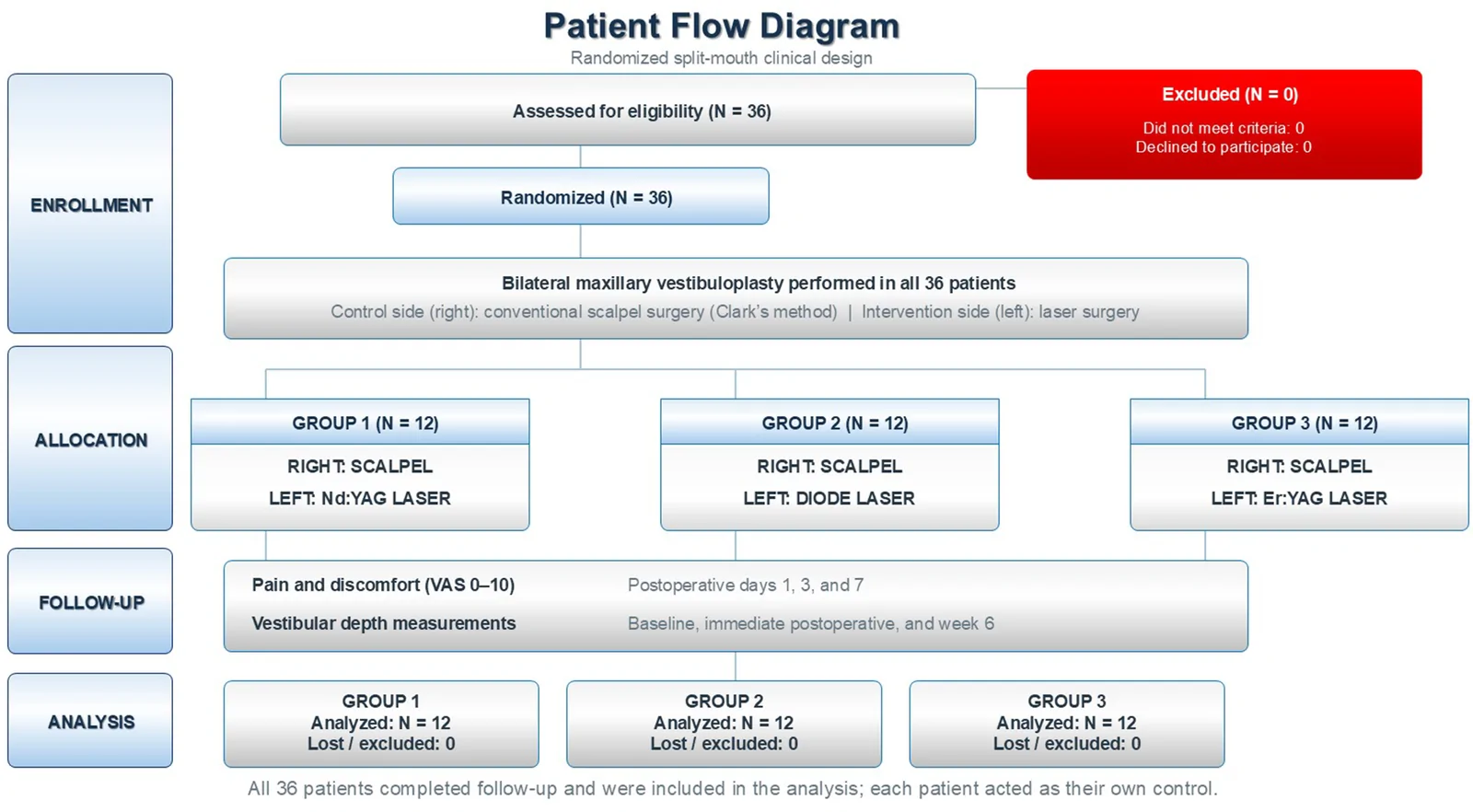
Patient flow diagram (CONSORT).

The patients were divided in 3 equal groups and the vestibuloplasty was performed by conventional surgery on the right side and by laser for the left side of the maxilla. The former served as a Control group. We utilized 3 types of lasers and consequently divided the patients in three study groups: for Group 1, the vestibuloplasty was performed by Nd:YAG laser, for Group 2 we utilized diode laser, while Group 3 was treated with Er:YAG laser (Figure 1).

An initial measurement of the vestibular depth was performed with the help of a millimeter markings William periodontal probe on three predetermined areas on each side: paramedian (next to the frenulum), antero-lateral (measured in the middle of the operated field), and lateral (at the most posterior place included in surgery). The measurements were performed 6 weeks postoperatively at the same levels as they were registered before surgery.

A visual analysis scales (VAS) was utilized for pain and discomfort assessment along the first, third and seventh day after the procedure on a range from 0 to 10. Score 0 was marked as “no pain” and “no discomfort”, while score 10 was “the most intense pain” and “the highest discomfort”.

The presence of bleeding at the end of the procedure on laser treated wounds was assessed clinically. We utilized the following scale for this aspect: Score 1 represents no bleeding, 2 represents self–limiting, while 3 requires a pressure to stop it. We must point out that it is well-know from the literature that the laser is preferred for its effective hemostasis and the unnecessary suture after surgical interventions. Therefore, we have chosen this simple scale to observe any minor differences in achieving hemostasis after laser application, as none of the interventions involved significant bleeding.

We closely analyzed the wound appearance at the end of the healing process.

All the participants were informed about the study protocol related to the conventional surgery and laser procedures, and their written consent was obtained.

### 2.3 Surgical protocol

**Figure 2:**
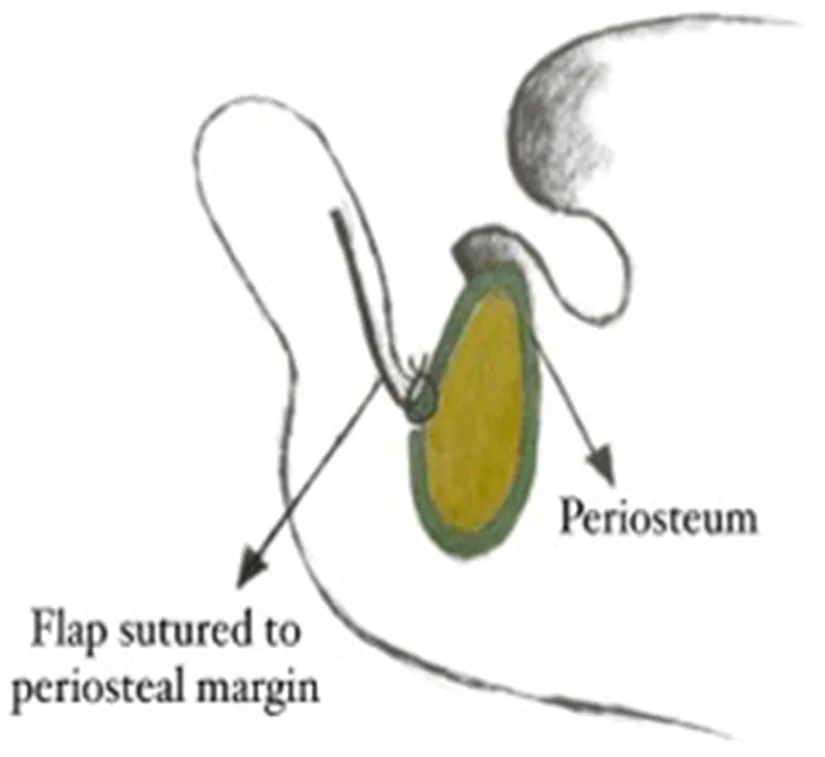
Schematic illustration of Clark^’^s vestibuloplasty.

Asepsis and infection control were followed prior to the surgery in accordance with the biosafety of norms. Clark's vestibuloplasty method was applied in all conventionally operated right sides of the patients. After the administration of local anesthesia with Articaine 1:100000, a horizontal incision was performed at the mucogingival junction using a scalpel blade 15C. This incision was made in the depth of the existing vestibule, following a trajectory parallel to the underlying maxillary bone surface to respect the anatomical configuration of the anterior maxillary region. A meticulous supraperiosteal dissection was subsequently performed, maintaining the periosteal integrity and allowing for a controlled separation of the mucosal and submucosal tissues from the underlying muscular attachments. The muscle insertions responsible for limiting vestibular depth were identified and progressively released from the periosteal surface by sharp and blunt dissection. The dissection was directed apically until an adequate mobilization of the labial tissues was achieved, with the complete elimination of muscular tension and the passive adaptation of the mucosa at the desired vestibular extension. The inner aspect of the labial mucosa was undermined to obtain sufficient flap mobility while preserving vascular supply. The repositioned pedicled mucosal flap was then stabilized at the newly established vestibular depth by the fixation to the periosteum using interrupted 4-0 absorbable sutures. This has allowed for a secondary epithelialization of the exposed periosteal surface and has minimized the risk of postoperative vestibular relapse caused by muscular reattachment (Figure 2). The wound healed in second intention without any additional graft.

For Group 1 (i.e., patients treated with the Nd:YAG laser), the procedure was performed under local anesthesia to ensure patient comfort and cooperation. The parameters for the Nd:YAG laser (Lightwalke, Fotona, Slovenia) that was utilized for this procedure were: center wavelength of 1,064 nm, output power of 4 W, frequency equal to 25 Hz, Micro Short Pulse (MSP) mode, sapphire fiber of 300 micrometers in diameter, power density equal to 6,229 W/cm^2^.

For Group 2 (i.e., patients treated with the diode laser), we utilized the Epic×^TM^ laser from Biolase, with a center wavelength of 940 nm and a pre-initiated tip, in a contact, continuous mode, with an output energy of 3 W. Ablation with the laser tip began at the mucogingival junction with a horizontal movement directing the laser parallel to the bone and thus slowly releasing the muscle fibers towards the new vestibule depth.

For Group 3 (i.e., vestibuloplasty performed with the Er:YAG laser), we utilized an Er:YAG laser (Lightwalker, Fotona, Slovenia) with a H14 handpiece, with the following parameters: center wavelength of 2,940 nm, contact mode, long pulse, energy of 200 mJ, frequency equal to 20 Hz; power of 4 W, water 2, gas 2.

For all the cases where the treatment was performed using lasers, suturing was not necessary because of the stability of the operated sites and of the bleeding control.

All the participants were previously informed about the complications that could occur: hemorrhage, infection, necrosis of the adjacent soft tissue or bone, scars, and risk of relapse (13). Post-surgical instructions were provided for all the cases, and the patients were prescribed antibiotics (Amoxycillin 875 mg and 125 mg clavulanic acid two times a day for 5 days) and analgesics (Paracetamol 500 mg two times a day for 2 to 3 days). A soft diet was indicated for the first week, and additional extraoral cold compressions were recommended for the first 2 days after the surgery.

All the patients were reviewed on days 1, 3, and 7. Also, they were examined for pain, discomfort, and clinical outcomes immediately postoperative and after 6 weeks, by using the VAS. A blinding of the study was performed, as the surgeon and the outcome assessor of the results have been different members of the team. On the other hand, the surgeon was the same for all patients, and the same for the outcome assessor.

**Figure 3:**
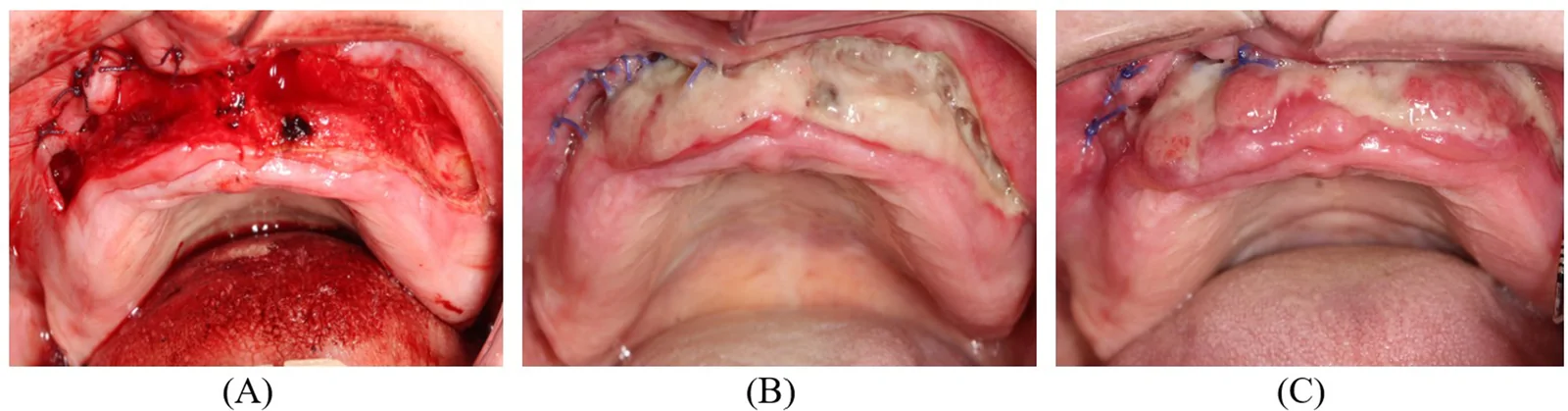
Postoperative examination of conventional versus Nd:YAG laser-based treatment (i.e., left versus right side of the image of the cavity): **(A)** immediately, **(B)** first day, **(C)** 6 weeks postoperative.

**Figure 4:**
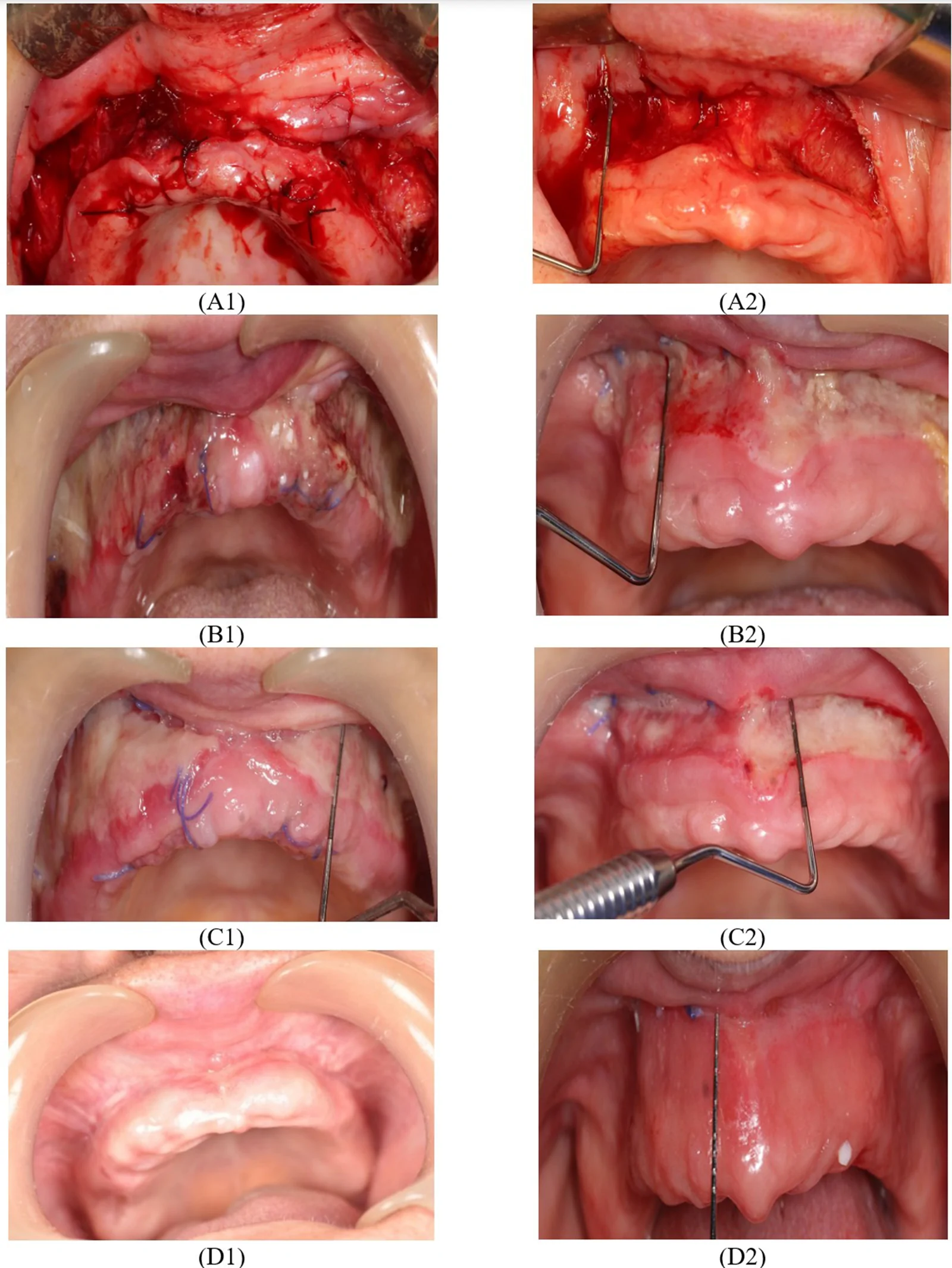
Postoperative examination of conventional versus (1) diode laser- and (2) Er:YAG laser-based treatment (i.e., left versus right side of the image of the oral cavity): **(A)** immediately, **(B)** first day, **(C)** 7 days, and **(D)** 6 weeks postoperative.

Standardized photography was performed to monitor the healing, and the new vestibule depth was measured at six weeks postoperatively. Examples of the patients’ evaluations in the comparison performed between conventional- and laser-based treatments are presented in Figures 3 and 4, for the three types of lasers considered in the study.

Further on, descriptive statistics were utilized to summarize the central tendency and dispersion of pain, discomfort, and vestibular depth evaluations. Data were reported as means and standard deviations (SD) or medians with interquartile ranges, depending on the distribution of values.

Primary and secondary outcome measures for the patients have included vestibular depth measurements that were further on compared between the study groups, both immediately (as primary outcome) and 6-week postoperatively (as secondary outcome).

## 3 Results

Pain and discomfort values were compared between the conventional surgery and the Nd:YAG laser, diode laser, and Er:YAG laser groups by using the Wilcoxon signed-rank test. The laser-treated sides have shown statistically significant reductions in both pain and discomfort scores at all considered timepoints (*p* < 0.05*)*, as presented in the following*.*

**Figure 5:**
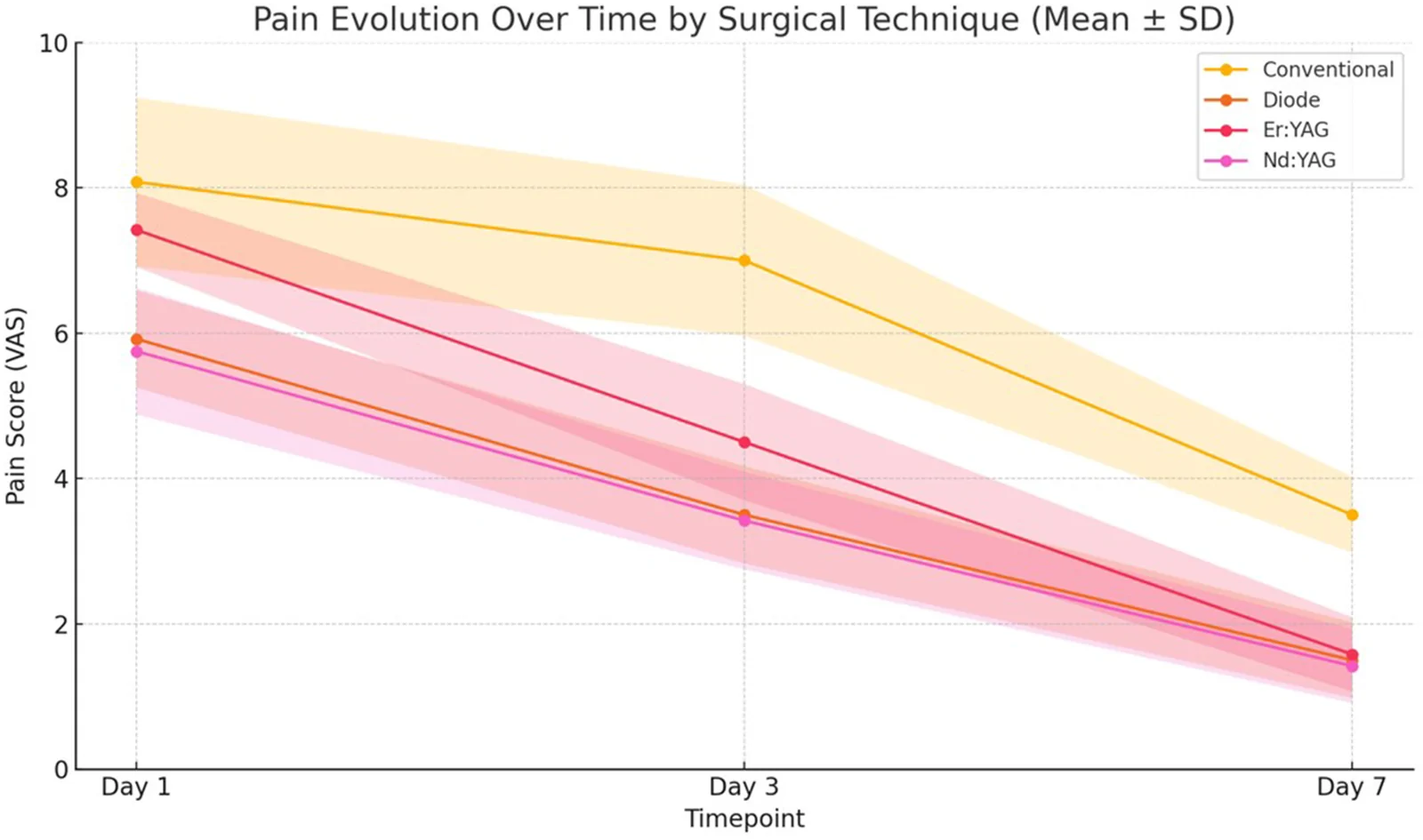
Pain evolution over time for each of the utilized surgical technique. Wilcoxon signed-rank test was utilized in the statistical analyses, with *p*-values < 0.001.

**Figure 6:**
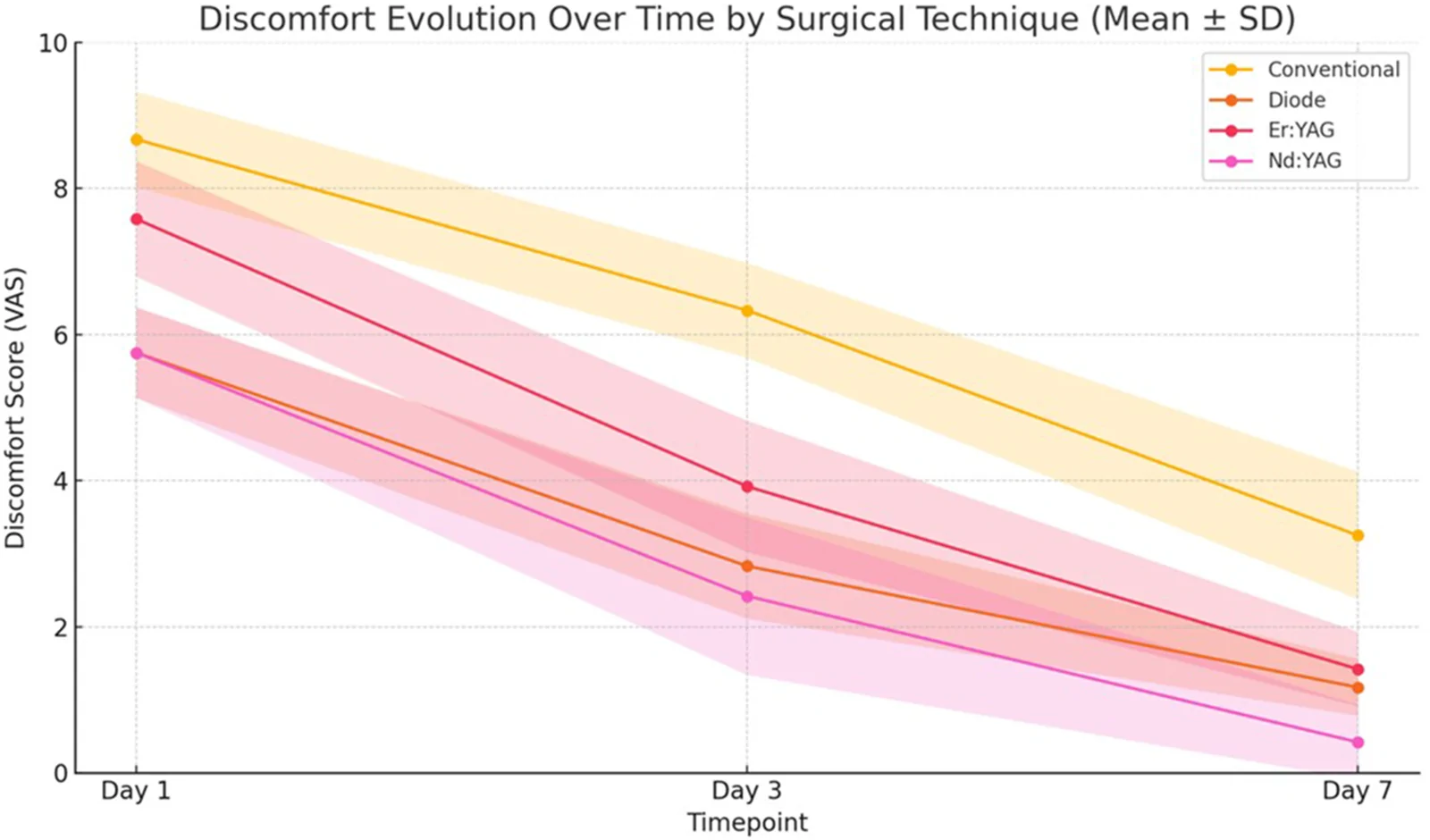
Discomfort evolution over time for each of the utilized surgical technique. Wilcoxon signed-rank test was utilized in the statistical analyses, with *p*-values < 0.001.

The evolution of pain and discomfort scores was assessed longitudinally using the VAS at days 1, 3, and 7 postoperatively for the four considered surgical techniques: conventional, diode laser, Er:YAG laser, and Nd:YAG laser. The mean values of the scores are presented in Tables 1 and 2, while the evolution of these values, including their SDs, is shown in Figures 5 and 6. Wilcoxon signed-rank tests were utilized to compare values between timepoints. Significant decreases were observed over time in both the conventional and the laser groups (*p* < 0.05), with faster resolution in the laser-treated sides. Across all the considered timepoints, the conventional surgery consistently produced the highest pain and discomfort scores, with mean values of 8.75 and 8.67 on day 1, respectively, and decreasing to 4.42 (pain) and 3.25 (discomfort) by day 7. In contrast, the performance of the Nd:YAG and diode lasers demonstrated the most favorable symptom profiles, with rapid and consistent reductions in both parameters. By day 7, the Nd:YAG laser resulted in the lowest mean scores for both pain (1.42) and discomfort (0.42), followed closely by diode (pain: 1.50; discomfort: 1.17). The Er:YAG laser showed higher initial values but comparable outcomes by day 7.

**Table 1:** Summative table for the postoperative evolution of pain.

Technique	Day 1	Day 3	Day 7	Total (Mean ± SD)
Conventional	8.75	6.75	4.42	6.64 ± 2.29
Diode	5.92	3.50	1.50	3.64 ± 2.29
Er:YAG	7.42	4.50	1.58	4.50 ± 2.94
Nd:YAG	5.75	3.55	1.42	3.57 ± 2.18

Wilcoxon signed-rank tests were utilized, with *p*-values < 0.001.

**Table 2:** Summative table for the postoperative evolution of discomfort.

Technique	Day 1	Day 3	Day 7	Total (Mean ± SD)
Conventional	8.67	6.33	3.25	6.08 ± 2.75
Diode	5.75	2.83	1.17	3.25 ± 2.49
Er:YAG	7.58	3.92	1.42	4.31 ± 3.24
Nd:YAG	5.75	2.42	0.42	2.20 ± 2.89

Wilcoxon signed-rank with *p*-values < 0.001.

These findings confirm that laser-assisted vestibuloplasty—especially using the Nd:YAG or the diode laser—offers superior postoperative comfort, accelerating symptom resolution compared to the conventional method.

Vestibular depth measurements were compared between the initial, the immediate postoperatively, and the 6-week postoperatively timepoints. Paired sample t-tests or Wilcoxon tests were utilized, depending on distribution, to evaluate changes within each technique (conventional and lasers).

**Figure 7:**
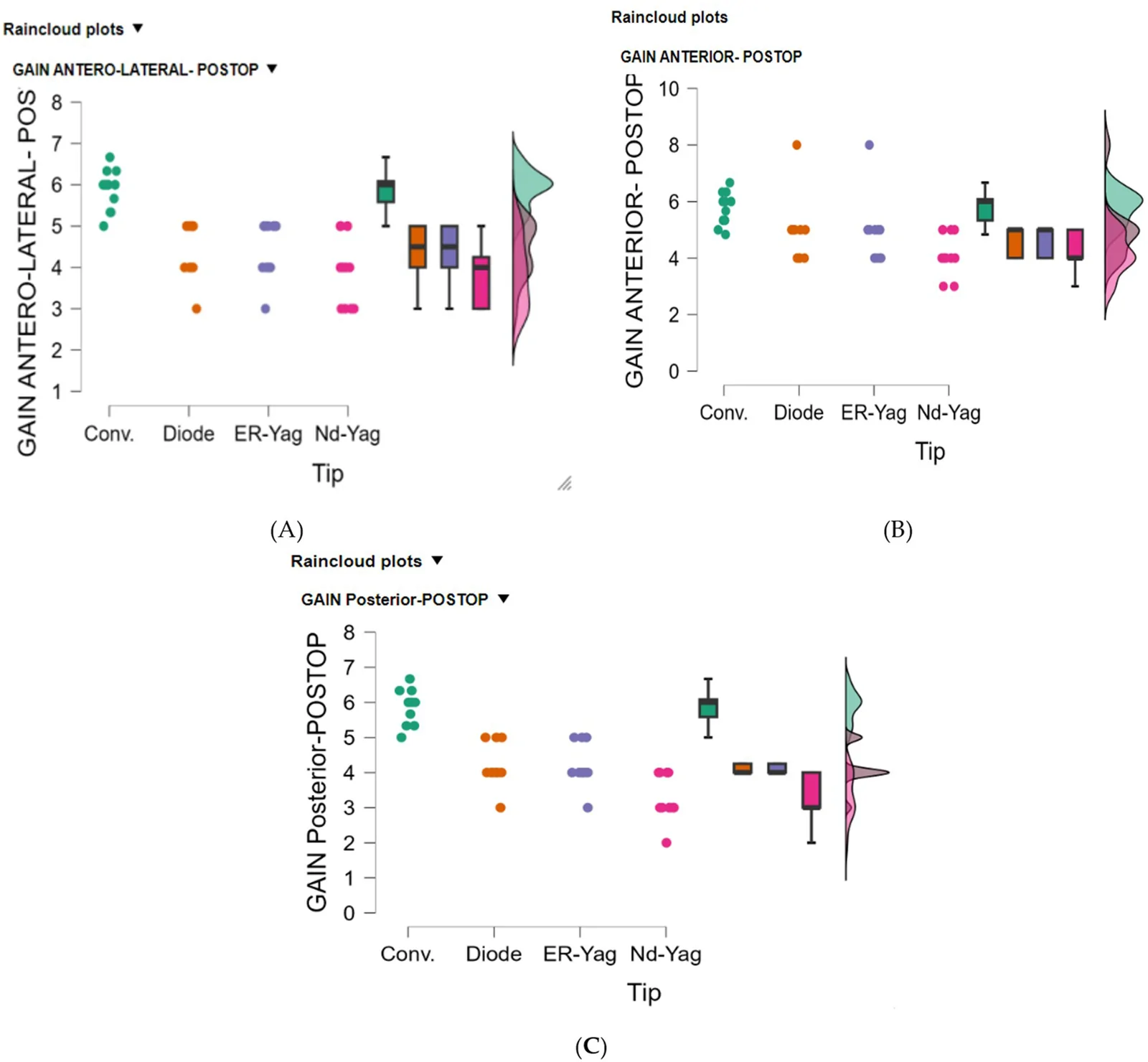
Measurements performed immediately postoperative by anatomical location: **(A)** antero-lateral; **(B)** anterior; **(C)** posterior. For the statistical analyses, antero-lateral and posterior, the one-way Kruskall-Wallis test was applied, while anterior, the one-way ANOVA test was applied (based on the distribution of the cohorts), in all cases with *p*-values < 0.001.

The vestibular gain was calculated as the difference between the 6-week postoperatively and the initial depth values. Further on, the vestibular gain was analyzed by anatomical location: anterior, anterolateral, and posterior (Figure 7). It can be observed that the vestibuloplasty performed by Nd:YAG laser achieved significantly higher gains in the anterior and posterior areas, while the contraction was reduced in the anterolateral zone compared to the conventional technique. Immediately postoperative, we obtained the best results in vestibule depth by conventional surgery in all the areas, while 6-week postoperatively the highest gain was achieved using the Nd:YAG laser.

**Figure 8:**
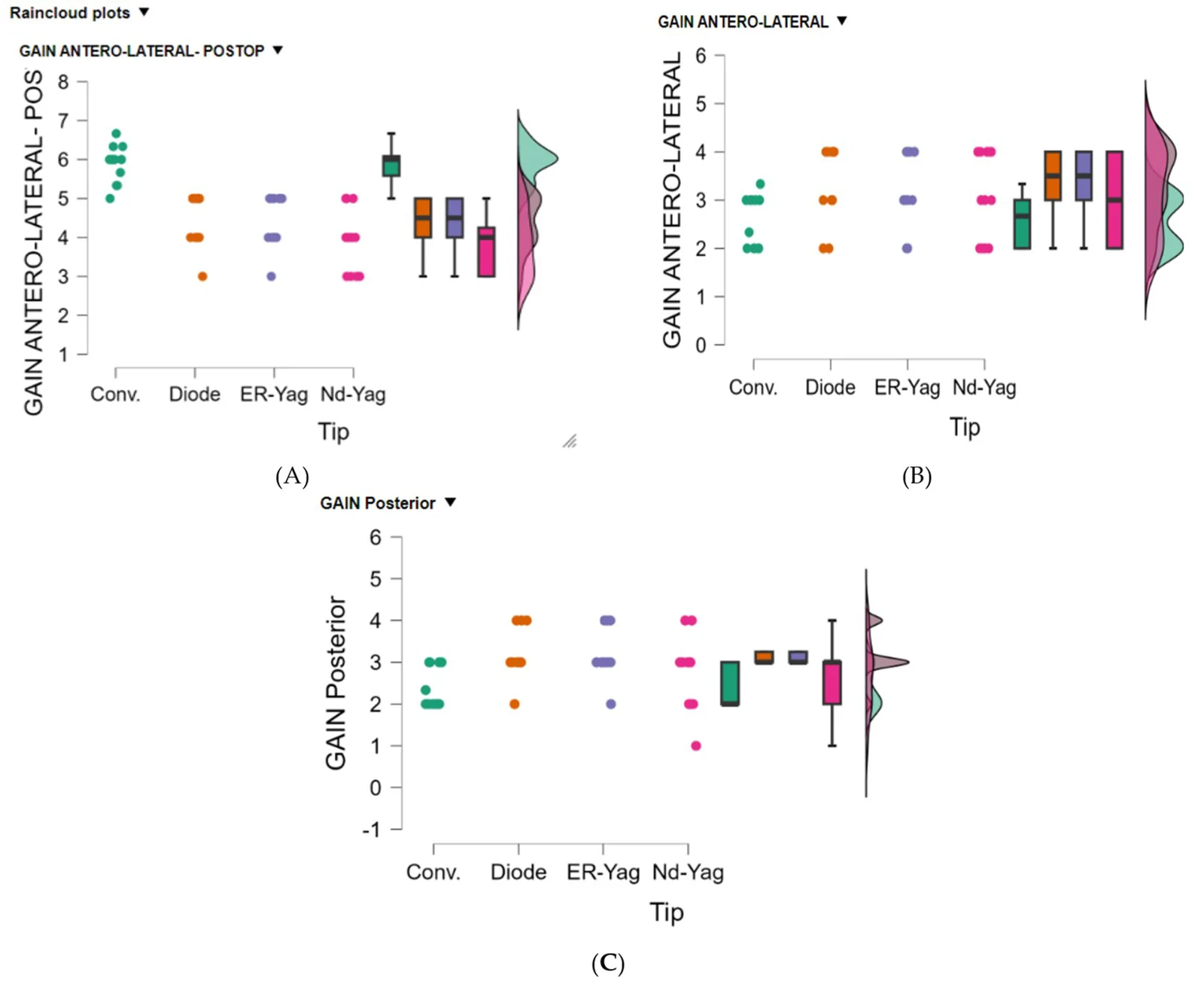
Gain in the vestibular depth by location: **(A)** antero-lateral postop; **(B)** antero-lateral; **(C)** posterior. For the statistical analyses, the one-way Kruskall-Wallis test was applied in all cases, with *p*-values < 0.001.

The Nd:YAG laser achieved significantly higher gains in the anterior and posterior areas, while contraction was reduced in the anterolateral zone compared to the conventional technique (Figure 8). Thus, comparative analysis between the conventional treatment and the Nd:YAG, diode, and Er:YAG methods showed a significantly higher gain in the laser groups, particularly in the anterior region (*p* < 0.05).

**Figure 9:**
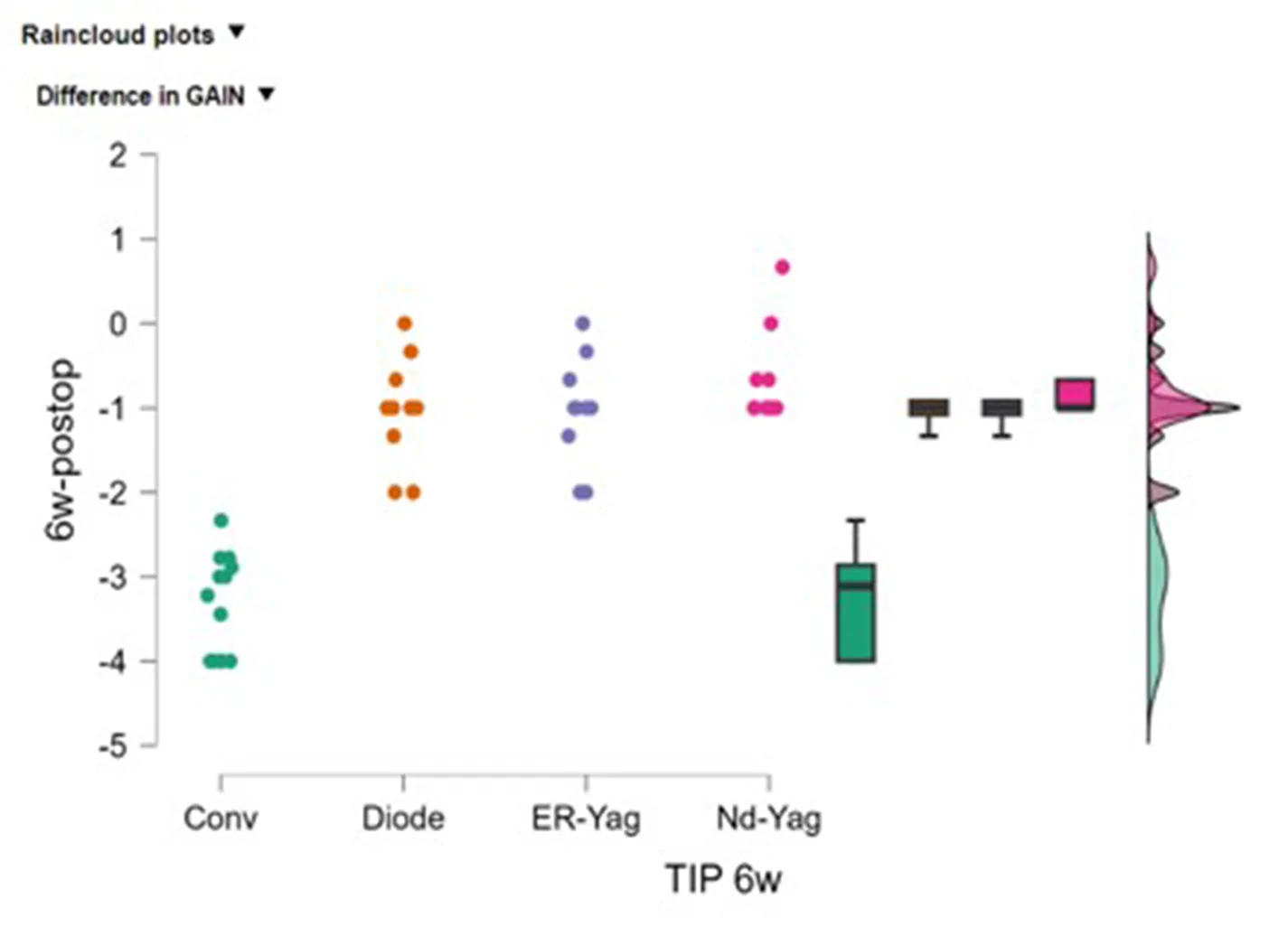
Vestibular depth obtained 6-weeks postoperatively. For the statistical analysis, the one-way Kruskall-Wallis test was applied, with *p* < 0.001.

The laser-treated sides have demonstrated more stable postoperative results with better long-term maintenance of vestibular depth (Figure 9).

Postoperatory bleeding was assessed on all the laser treated sides. A self-limiting bleeding was noticed when Er:YAG laser was utilized, level 2 on our scale.

**Figure 10:**
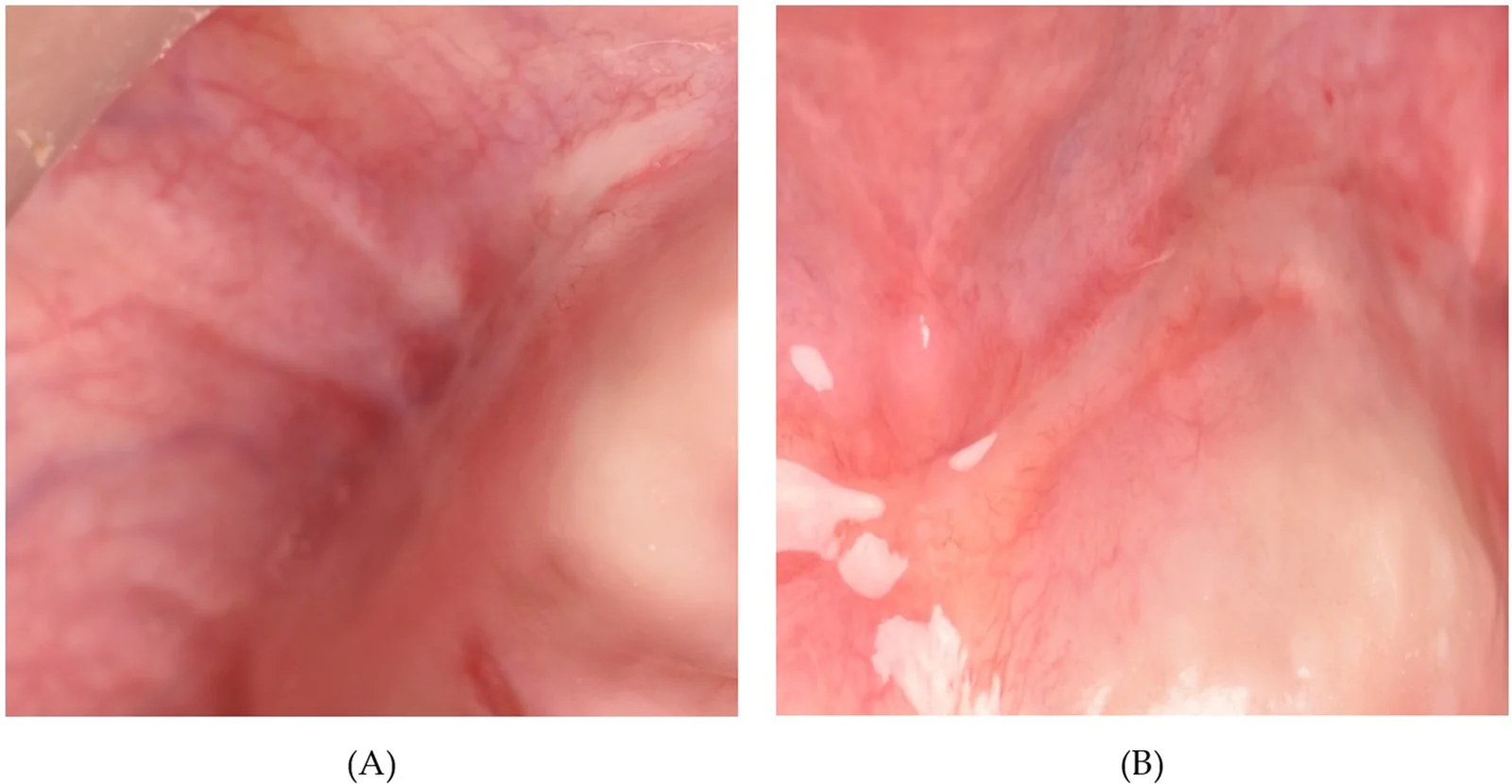
Magnified healing areas obtained using **(A)** Clark’s technique versus **(B)** laser surgery.

The healing areas were closely examined. The regions healed through secondary epithelialization were rough, irregular and unequal, while the laser-treated sides were flat and smooth (Figure 10). We must highlight that the wound aspect after healing is important when it interacts with the margins of the prosthesis in an intimate contact over a tender underlying periosteum. A flat and smooth scar obtained in the laser-treated patients ensures prosthesis-wearing comfort and facilitates its adaptation to constant pressure.

## Discussion

### 4.1 Conventional vestibuloplasty

Today, the use of laser in oral surgery brings a new performance environment due to the major change achieved with regard to conventional approaches. Better clinical results and smooth perception from the patient^'^s point of view have been obtained. The scope of this study, i.e., to assess and compare the results of performing vestibuloplasty by scalpel and lasers, was accomplished. In the considered clinical cases we determined whether the laser assisted vestibuloplasty could be a better option of treatment that is capable to achieve and maintains a stable vestibular depth.

Despite the advances of contemporary dental treatments, many people still require teeth replacement by total prostheses. This is the main reason why vestibuloplasty is returning today in the oral surgeons and implantologists focus because of the high demand of attached gingiva around dental implants. Moreover, the edentulous arches require stability and retention for the total prostheses. This is also provided mostly by a stable keratinized tissue. The majority of edentulous patients often experience multiple health conditions that can significantly impact their healing capacity. Consequently, less invasive procedures with a reduced recovery time, less pain, and no complication must become a standard for interventions.

The currently utilized conventional vestibuloplasty is an invasive procedure for elderly patients. It causes bleeding, postoperative pain and discomfort, while it requires a prolonged operatory time because of the necessity of suture. The literature describes three types of techniques, in accordance to the bone level and to the quality of soft tissue (14):

- The submucosal advancement technique is utilized in cases with sufficient bone and in the presence of healthy soft tissue. This technique can be performed by submucosal and supraperiosteal dissection in a closed approach (as performed initially by Obwegeser in 1959) or in an open view approach (as developed by Walleneus in 1963).
- The secondary epithelialization-based techniques performed in cases with sufficient bone but poor soft tissue quality to start with. It was initially performed by Kazanjian in 1924, using a mucosal flap from the inner aspect of the lip, undermined it in a submucosal fashion, and sutured it to the intact periosteum. The drawback is leaving a scarring lip with decreased mobility and impaired function (15). The technique was modified by Godwin, Lipswitch and Mejchar, related to the crestally pedicled flap creation, to achieve a better vestibule depth and to maintain the obtained results. Clark created a lip pedicled flap, a reverse of Kazanjian’s technique, which created a crestal pedicle flap. The methods of gaining depth through secondary epithelialization have the disadvantage of scar contracture with a gradual loss of the result.
- The grafting is performed in cases with insufficient bone or with large bone grafting procedures. The grafting materials are mostly autogenous mucosal tissue, but also allograft tissues such as acellular dermal matrix and xenogeneic collagen matrix. These added materials bring advantages that include less relapse, early covering of the surgical wound, and more rapid healing (16).

Apically positioned flap combined with free gingival graft is considered to be the most effective method for acquiring attached gingiva (11, 17). Some previous studies were performed using skin grafts and major beneficial effects were found after vestibuloplasty. Thus, the method increases the mechanical resistance to displacement and enhances the stability of the denture. Also, skin grafts, which tend to form hyperkeratosis, diminish the touch perception and make the wearing of a prosthesis more comfortable. Last, but not least, because of the decreased trauma to the bone, this type of procedure slows the bone resorption (16). Today, in order to reduce the morbidity by harvesting skin and to eliminate the patient's dissatisfaction related to bad taste and odor, the free skin graft was replaced by gingival graft, which is characterized by a better patient assessment (18).

Although secondary intention healing resulted in clinically stable and keratinized-appearing tissue in all treated cases, the most predictable method for obtaining a well-defined zone of keratinized tissue remains the use of a free gingival graft harvested from the palatal mucosa. Such a graft provides a histologically keratinized epithelial surface with a well-established connective tissue component and remains a reference technique when a substantial increase in the width of keratinized mucosa is required. However, this approach involves an additional surgical site, an increased operative time, a greater postoperative morbidity, and a potential discomfort associated with palatal harvesting. Also, it could have led to an unsatisfying result, because of the excessive mobile tissue present at the recipient site or because of the poor fixation of the graft. Combining vestibuloplasty with allografts or xenografts for the patients targeted in our study would have increased the costs. Also, the time-consuming procedure would not have been in accordance to our patients'^,^ expectations. These considerations are particularly relevant in elderly patients, in whom minimizing surgical complexity and reducing postoperative morbidity may represent important clinical objectives.

Therefore, the approach utilized in the present study was selected as a less invasive alternative aimed at increasing vestibular depth while allowing for the development of a clinically stable mucosal surface without the need for a second donor site. Nevertheless, we acknowledge that histological confirmation was not performed and that free gingival grafting remains the most predictable approach when the primary objective is the augmentation of a clearly defined band of keratinized tissue.

Therefore, Clark^'^s technique was utilized in our study for all the conventional surgery sites. It is a technique suitable for cases where the height of the bone is adequate, but the soft tissue is of poor quality, as we have had in all our cases. This technique of vestibuloplasty through secondary epithelialization consists of creating a split lip pedicled flap, elevated submucosally and supraperiosteal which is sutured at the base of the new vestibule with absorbable sutures. Clark^'^ s method of gaining depth through secondary epithelialization has a major disadvantage, consisting of a scar contracture with a gradual loss of the result (19).

### 4.2 Rationale for laser use

Vestibuloplasty can be performed with lasers such as carbon dioxide (CO_2_), diode, Er:YAG, and Nd:YAG (20).

*The diode laser* had been utilized in one of our study groups because of its advantages, i.e., comfortable size and affordability, which make it the most utilized alternative device in oral surgery. It has already demonstrated its advantages in vestibuloplasty: hemostatic and antibacterial properties, flexibility, and accessibility in posterior areas due to its small active part (21–23). It is already demonstrated that high-power diodes such as those with a 940 nm center wavelength that we have utilized have the potential to distribute light to more profound tissues and thus, to add photobiomodulation (PBM) to the cutting effect (24). *In vitro* studies show that low-energy light is absorbed in mitochondria through chromophores. Therefore, cell proliferation through photochemical alterations is increased. This mechanism adds promotion of angiogenesis, collagen production, proliferation and differentiation of osteogenic cells, mitochondrial respiration, and synthesis of adenosine triphosphate (ATP) (25). These effects increase the cell metabolism and reduce the inflammatory processes in the irradiated areas. This goes beyond a stimulatory reaction of the local immune response. All of these, taken together, allow the tissues to act better to negative and potentially harmful local stimuli (26, 27).

*The Er:YAG laser* that operates at a center wavelength of 2,940 nm has a high absorption in water. It yields advantages that include reduced anesthesia requirements, diminished postoperative sensitivity, and decreased swelling (28). By using Er:YAG lasers, tissue ablation is performed in narrow and precise layers with low thermal damage (29), conducting to minimum pain and discomfort, as we have obtained for our operated patients. Er:YAG laser is assigned in literature as not suitable for oral soft tissues surgery because of the lesser hemostatic effects (30). Our results support this conclusion, as we had a self-limiting bleeding in all the cases where Er:YAG was utilized in comparison to the cases treated with diode and Nd:YAG lasers, for which no bleeding occurred. However, from our point of view, even with a prolonged bleeding immediately after the surgery, we consider the Er:YAG laser a suitable instrument for vestibuloplasty, because of the regular incision that is comparable to the one performed with a scalpel and also because of the reduced thermal effects, which has the clinical significance of low pain and discomfort.

*The Nd:YAG laser* is particularly absorbed by hemoglobin. At a wavelength of 1,064 nm, because of its poor absorption in water, it penetrates deeper into the tissue (i.e., about 4 to 5 mm), with a photocoagulation effect extended up to 7, and even to 10 mm (31). Previous works demonstrated that when the Nd:YAG laser is utilized for intraoral lesions, it provides low rates of pain and edema, a good bleeding control, and no damage to the surrounding tissue (32, 33). These properties of Nd:YAG have served for our cases the effectiveness in vestibuloplasty in terms of hemorrhage control and stability of new vestibule depth. These have proved to have the highest values in comparison to the other lasers, as well as to the conventional method, as demonstrated by the results shown in Figures 7–9.

*Conventional vestibuloplasty* was perceived by our patient as more painful and rough, especially in the first days after surgery, compared to the laser-treated sides. Conventional surgery has consistently produced the highest pain and discomfort scores on day 1, decreasing to a half of the symptoms in the third day, but still present even by day 7. In contrast, the Nd:YAG and diode lasers have demonstrated the most favorable symptom profiles, with rapid and consistent reductions in both parameters. By day 7, the Nd:YAG laser resulted in the lowest mean scores for both pain (1.42) and discomfort (0.42), followed closely by the diode laser (pain: 1.50; discomfort: 1.17), as presented in Tables 1, 2. The Er:YAG laser showed higher initial values but comparable outcomes by day 7.

These findings confirm that the laser-assisted vestibuloplasty (especially with Nd:YAG or diode lasers) offers superior postoperative comfort by accelerating the symptom resolution, in comparison to conventional methods. These results agree with previous studies that also demonstrated the patients' comfort in diode laser groups, because of the activation and manufacture of endogenous analgesics as well as because of a decrease in C-fiber neural activity (34). The Nd:YAG laser utilized in vestibuloplasty has already demonstrated its supremacy because of its minimum tissue absorption and of its maximum penetration (35).

The present study possesses several distinctive methodological and scientific strengths that, to the best of our knowledge, make it one of the few comprehensive comparative investigations in the field of laser-assisted vestibuloplasty. Unlike previous reports, which predominantly focused on single-laser approaches or comparisons between diode lasers and conventional scalpel techniques, the current study has simultaneously evaluated three different laser systems, in direct comparison to the conventional scalpel vestibuloplasty, under standardized clinical conditions. Furthermore, the study employed a split-mouth design. This has allowed each patient to serve as their own control, thereby minimizing interindividual variability and significantly increasing the internal validity and reliability of the obtained results. This methodological approach provides a highly accurate assessment of postoperative outcomes and tissue response associated with each surgical modality. In addition, the inclusion of 36 patients represents a comparatively large sample size for a clinical study of this nature, particularly within the domain of oral laser surgery, where most available studies are limited to small case series or pilot investigations. Collectively, these aspects confer meaningful evidences regarding the clinical effectiveness and biological behavior of different laser technologies in vestibuloplasty procedures.

### 4.3 Comparison of clinical outcomes

In our study we have monitored the healing. Therefore, we have also noticed the loss in height of the new vestibule obtained through conventional surgery in comparison to laser-assisted vestibuloplasty sides, which were more stable during the maturation phase. This important aspect can be explained by the wound contraction induced by the healing process (36). The secondary epithelialization which occurs in Clark^'^s vestibuloplasty has the same healing process as an excisional wound. As a consequence, during healing the newly attached mucosa becomes loose and allows for the recently disinserted muscles to reattach into preoperative levels, causing relapse (37). Previous studies demonstrated that an oral mucosa wound induced by laser heals by fewer contractile myofibroblasts compared to a scalpel wound, which heals by contraction (38, 39). The laser-treated sides demonstrated more stable postoperative results, with better long-term maintenance of the vestibular depth compared to the conventionally-operated sides. We must also highlight one of the major advantages of laser surgery over conventional scalpel, i.e., be to reduce the risk of bleeding in medically compromised patients on Anticoagulant medication (40).

By comparing the results obtained using the three considered lasers, we may conclude that the Nd:YAG laser has offered the best balance of depth gain and comfort, as presented in the Results section and discussed in the previous subsection.

However, for a balanced discussion, we must also point out to limitations that include contraindications of laser use, or variability in operator skill. For example, Er:YAG's known lack of coagulation is an issue that requires a critical risk-benefit analysis.

Due to the fact that we applied the laser only on edentulous prosthetic field, the risk of adjacent teeth damage was completely eliminated, as well as the risk of periodontal ligament necrosis, ankylosis, or cementum resorption. From the literature, these are the most encountered undesired effects in laser therapy (41).

In the present study, suturing was performed only in the scalpel vestibuloplasty group, as this technique involved conventional mucosal incision and flap repositioning that required the stabilization of the surgical margins at the newly established vestibular depth. Conversely, the laser-assisted vestibuloplasty group was intentionally left unsutured because the laser procedure was performed without conventional flap elevation or mucosal repositioning. This has allowed for a spontaneous healing of the treated surface. We acknowledge that the presence of sutures may represent a potential confounding factor when comparing the considered techniques, as sutures may contribute to the mechanical stabilization of the repositioned tissues and could theoretically influence postoperative contraction and vestibular relapse. Therefore, the differences observed between the two approaches should be interpreted considering not only the effect of the surgical instrument itself but also the distinct wound management protocols associated with each technique.

This study has confirmed the hypothesis that laser treatments offer a better clinical outcome for the patients compared to conventional methods in what concerns preprosthetic vestibuloplasty. Wound healing is also improved through the formation of a proteinaceous layer that serves as a protective barrier. In the oral cavity it protects the wound from both bacterial contamination and mechanical irritation, the latter caused by the masticatory process (42).

The proposed reduction in scar contraction following laser surgery is based on the hypothesis that a more controlled and superficial tissue injury may influence the early inflammatory and remodeling phases of wound healing. Unlike the conventional scalpel vestibuloplasty, which involves mechanical incision, tissue dissection, muscle attachment release, and subsequent wound stabilization, the laser-assisted vestibuloplasty produces a controlled photothermal effect that is restricted to the targeted mucosal and superficial connective tissue layers. This may result in reduced collateral tissue trauma and a more limited inflammatory response. The biological rationale is that excessive inflammation and prolonged activation of fibroblasts may promote differentiation into contractile myofibroblasts, which are key cellular mediators of wound contraction through *α*-smooth muscle actin expression and extracellular matrix remodeling. By potentially reducing tissue disruption, bleeding, and inflammatory mediator release, laser surgery may create a healing environment characterized by a more regulated fibroplastic response and reduced excessive collagen contraction. Thus, laser surgery reduces scar contraction by creating a controlled, superficial injury that limits collateral damage and alters the early inflammatory response compared to conventional methods. This modulation minimizes the differentiation of fibroblasts into myofibroblasts by downregulating Transforming Growth Factor-beta 1 (TGF-*β*1), resulting in a more organized collagen structure and reduced contracture (43).

However, we acknowledge that direct evidence that demonstrates a reduced myofibroblast activity specifically after laser vestibuloplasty remains limited. Therefore, this mechanism should be considered a biologically plausible hypothesis rather than a definitive explanation of the clinical findings. Therefore, we emphasize that the observed reduction in postoperative contraction may result from the combination of controlled tissue injury, improved hemostasis, reduced inflammatory burden, and altered wound remodeling rather than from a single biological pathway.

Regarding the comparison between the two types of techniques, for a more balanced view, one must point out that the cost and equipment accessibility are in favor of conventional methods. The potential learning curve may also be an issue for medical doctors, and efforts are made in medical schools (as well as through training stages) to accommodate practitioners with laser techniques. Cases where lasers might not be indicated may be another issue, and they include issues specific to laser general safety regulations, smoke and vapor evacuation control, and the risk of fire or explosion when using alcohol-based disinfectants (44).

### 4.4 Limitations and future research

Limitation of our study includes considering only elderly patients, although this was imposed by the specific issues of such patients, as discussed above. The limited number of patients included in the present study was another aspect that is planned to be addressed with further clinical studies in our group. We must also point out limitations like monocentric design, subjectivity in bleeding and wound healing assessment, as well as lack of blinding. The lack of a standardized protocol for each type of laser and its assigned procedure may lead to limitations in the study’s results, as well.

In the present study, immediate or early reinsertion of the prosthesis was not performed because the existing prosthesis had not been previously adjusted or relined to accurately correspond to the newly established vestibular anatomy. Therefore, its immediate use could have resulted in a non-controlled pressure on the surgical site, potentially interfering with the wound stabilization, the mucosal adaptation, and the accuracy of the postoperative vestibular assessment. The decision has been made to allow for an undisturbed healing without a prosthetic pressure during the initial healing phase. This aimed to obtain a reliable evaluation of the surgical outcome and the actual vestibular depth achieved by the Clark vestibuloplasty procedure. The postoperative protocol consisted of careful wound monitoring and maintenance of oral hygiene measures according to the study protocol, while avoiding mechanical irritation of the healing tissues.

We acknowledge that an immediate or early adapted prosthesis may serve as a mechanical stent, supporting mucosal positioning, maintaining vestibular depth, and potentially reducing postoperative scar contraction. However, this approach requires a properly adjusted prosthesis or a prefabricated surgical stent that provides a controlled support without an excessive pressure on the healing tissues. Since such a customized prosthetic device was not available in our protocol, the prosthetic reinsertion was intentionally avoided.

Potential directions of research along the same lines may include considering other types of patients for a comparison of these (laser-based versus conventional) procedures. Also, we clearly envisage in general the need for long-term studies (i.e., longer than 6 months) to assess the durability of the obtained effects. However, for the type of patients considered in the present study, we have observed that a long-term follow up cannot be scientifically sound. The results of such a long-term study could not be accurately interpreted due to the addition of factors that progressively alter the prosthetic field over time, including: the continuous and consistent bone remodeling caused by ongoing alveolar resorption; the quality of the mucogingival phenotype, which influences the individual rate of resorption; the dental occlusion, which is often unbalanced in elderly individuals, most frequently due to improper mandibular prosthetic rehabilitation and bruxism.

As a previous study demonstrated an impact of gender in the perception of pain, such comparisons between patients would be of interest when studying differences in the VAS scores (45).

Regarding the performed analyses, for the present study we split on the midline the treatment type with no possibility of crossover healing due to the anterior nasal spine where the prominent crest and the thickened periosteum divide very steep the treated territories. Thus, we have not initiated histological analysis because the vestibuloplasty was performed on healthy tissue without any biological material removal.

Combination of laser vestibuloplasty with autogenous materials can be another further direction for research. They could add a real value to conclusions because of aspects such as a minimum tissue trauma, the capability to promote a faster healing, and a lower risk of immune rejection or inflammatory response.

As previous studies have utilized Low Level Laser Therapy (LLLT) for further relief of post-operative pain, such aspects are also beneficial to be considered in future studies, as LLLT produces a release of *β*-endorphins and a decrease of the activity of C fibers (46).

Another necessary direction of work refers to the training of medical students and practitioners, as we have approached for other clinical procedures, as well (47).

## 5 Conclusions

This study has performed a comparison between conventional and laser vestibuloplasty, considering three types of lasers, i.e., Nd:YAG, diode, and Er:YAG laser. We demonstrated that laser-based treatments are superior in what concerns the clinical outcomes evaluated immediately postoperative, as well as after 1, 3, and 7 days, as well as 6 weeks after the treatment. Several aspects have been evaluated and analyzed, i.e., pain, discomfort, and bleeding, all using a VAS. The vestibular depth was measured and the vestibular gain was determined and analyzed to assess the efficiency of each type of laser type for the considered clinical procedure. The advantages and drawbacks of each laser treatment was discussed. We may conclude that the positive aspects associated with the use of lasers in preprosthetic vestibuloplasty have increased the patients' compliance with the treatment and therefore lasers appear to offer clinical advantages in specific settings for such clinical procedures. By comparing the results obtained using the three considered lasers, we may conclude that the Nd:YAG laser offers the most stable results over the healing time. Regarding pain and discomfort, Nd:YAG and diode lasers demonstrated the most favorable symptom profiles, with rapid and consistent reductions in both parameters. The Nd:YAG laser has offered the best balance of depth gain and comfort.

## Data Availability

The original contributions presented in the study are included in the article/Supplementary Material, further inquiries can be directed to the corresponding author.
